# Mechanical Behavior of Liquid Nitrile Rubber-Modified Epoxy Resin under Static and Dynamic Loadings: Experimental and Constitutive Analysis

**DOI:** 10.3390/ma11091565

**Published:** 2018-08-30

**Authors:** Xiao Xu, Shiqiao Gao, Zhuocheng Ou, Haifu Ye

**Affiliations:** 1 State Key Laboratory of Explosion Science and Technology, Beijing Institute of Technology, Beijing 100081, China; gaoshq@bit.edu.cn (S.G.); zcou@bit.edu.cn (Z.O.); 2 Institute of Electronic Engineering, China Academy of Engineering Physics, Mianyang 621900, China; yehaifu@163.com

**Keywords:** LNBR/epoxy composites, compression experiments, ZWT nonlinear viscoelastic model, incremented algorithm

## Abstract

Quasi-static and dynamic compression experiments were performed to study the influence of liquid nitrile rubber (LNBR) on the mechanical properties of epoxy resin. The quasi-static experiments were conducted by an electronic universal machine under strain rates of 0.0001/s and 0.001/s, while a Split Hopkinson Pressure Bar (SHPB) system was adopted to perform the dynamic tests for strain rates up to 5600/s. The standard Zhu-Wang-Tang (ZWT) nonlinear viscoelastic model was chosen to predict the elastic behavior of LNBR/epoxy composites under a wide range of strain rates. After some necessary derivation and data fitting, a set of model parameters for the tested materials were finally obtained. Meanwhile, the incremented form of the ZWT nonlinear viscoelastic model were deduced and implemented into the user material program of LS-DYNA. A simulation-test contrast had been performed to verify the validity and feasibility of the algorithm. The results showed that the viscoelastic behavior of epoxy resin can be effectively simulated.

## 1. Introduction

High strength, electrical insulation and chemical stability enable the epoxy resins to be widely used in many fields, including military and civilian industries. In order to extend their application areas, the mechanical properties of epoxy resin are modified using various methods. During material preparation, the mechanical behavior of the epoxy resins can be changed through the manufacturing process. In addition, many additives can be blended with epoxy resins to obtain different performance of the epoxy materials.

It has been reported that the thermal treatment can help to modify the mechanical behavior of the polymeric materials. Post-curing and thermolysis are two competitive processes during temperature exposure [1]. The former one refers to the heat treatment above the glass transition temperature of a cured polymer, which can increase the degree of crosslinking by about 20–30% and elevate the stiffness as well as strength of the material. Nevertheless, the thermolysis is considered to appear along with the beginning of heat treatment process, which leads to the decrease in density of intramolecular crosslinks and reduce the shear modulus accordingly. Consequently, Mlyniec et al. [1] presented a structurally based constitutive model, which took the influence of the post-curing as well as thermolysis process on stiffness of epoxy adhesives into account.

Epoxy resins are often blended with various fillers which have been extensively researched to improve their physical and mechanical properties. Common fillers include fibers, rigid-particles, rubber particles and so on. Some researchers mentioned that carbon fibers could enhance the fracture toughness of epoxy resin [2] and the orientation of carbon fibers could also help to improve the stiffness of the fiber/epoxy composites [3]. Some other researchers suggested that rigid particles could improve the fracture toughness, stiffness and compression strength of the epoxy resin [4,5]. In addition to fibers and rigid particles, soft particles were also applied as a reinforced phase of epoxy resin. The technique known as “rubber-toughening”, which mixes soft rubber particles with epoxy resin, can be adopted to increase the toughness of the matrix material. The toughening effect is caused by the blocks generated from the reaction between the reactive end groups of the rubber particles and the active group of the epoxy resin [6]. The blocks will precipitate from the matrix during the curing process of the resin to form a two-phase structure. Afterwards, the second phase (rubber) can induce the energy dissipation process in the matrix, which enhances the resistance to plastic deformation of the matrix material [7]. Fakhar et al. [8] chose vinyl-terminated butadiene acrylonitrile (VTBN) rubber as a toughening agent and conducted a series of contrast tests. The results showed that the tensile elongation, mode I fracture toughness, impact strength and energy absorption capability of the modified epoxy were elevated compared with the pure epoxy resin. Liu et al. [9] found that soft rubber nanoparticles could increase the fracture energy of epoxy more than hard nanosilica. Thus, this technique has been used to increase the fracture resistance and fatigue life of epoxy resin [10]. However, it has also been reported that soft nanoparticles may reduce the Young’s modulus and yield strength of the composite at the same time [8,9,11]. This is because that the soft rubber particles act as stress dissipaters to decrease the yield strength of the resin.

As is well-known, amorphous polymers in the glass state exhibit either viscoelastic-viscoplastic or brittle behavior and many of those polymers are in the glass state at room temperature. Namely, those polymers and their composites present viscoelastic-viscoplastic properties at room temperature. Constitutive models for describing the mechanical behavior of polymers have been introduced in many published literatures. An elasto-viscoplastic constitutive model consisting of 13 parameters was developed to model the elasto-viscoplastic behavior of the thermoset epoxy for different temperatures and different quasi-static loadings [12]. This model accounted for temperature-dependent elastic moduli, yielding and hardening prior to the peak stress, as well as quantitative representations of strain rate and temperature sensitivity, which could correctly represent the behavior of materials under different temperatures and quasi-static strain rates. The Bergstrom-Boyce model [13] is a typical hyperviscoelastic constitutive model, which can be adopted to describe the hyperelasticity and viscosity of the polymer. The model can be divided into two parts: a hyperelastic model and a time-dependent Maxwell model. This model can describe the hyperviscoelastic behavior of rubber with a strain below 0.8 and strain rate below 1/s. Boyce et al. [14] also developed a new physically based constitutive model, which could capture the transition in the yield behavior and accurately predicted the post-yield, large strain performance of the polymer over a wide range of temperatures and strain rates. Sun et al. [15] used a simple constitutive model to represent the elastic/viscoplastic behavior of composites. With this model, creep and relaxation effects could be considered and predicted. Kontou [16] adopted a viscoplastic model, which consisted of a nonlinear Maxwell model in parallel with a Langevin spring, to describe the tensile and stress-relaxation behaviors of epoxy resin. Nevertheless, the above models either require too many parameters which are not suitable for engineering applications, or cannot reflect the impact response of polymers under high strain rates. Jiang et al. [17] built a new modified Zhu-Wang-Tang (ZWT) nonlinear viscoelastic constitutive model which could represent the compression behavior of ethylene propylene diene monomer (EPDM) under different strain rates varying from 0.0001/s to 0.001/s. A thermal-viscoelastic ZWT constitutive model was proposed by Zhang et al. [18] to describe the tensile response of the polyurethane interlayer under a wide range of strain rates and temperatures. Fard et al. [19] built a multilinear stress-strain model to describe the tensile and compression behavior of epoxy resin materials under large deformation. 

In addition to building constitutive models, some researchers also implemented these models into some finite element (FE) softwares to simulate the mechanical behavior of the polymers. A three-dimensional constitutive model was derived by Iwamoto [20] based on a four-element model with an elastic series element, which was implemented into the user subroutine user defined material subroutine (UMAT) of ABAQUS/Explicit to simulate the quasi-static and dynamic compression behavior of carboxy-terminal butadiene-acrylonitrile(CTBN)-modified epoxy resin. Lee et al. [21] proposed a unified anisotropic elasto-viscoplastic damage model and the relevant computational analysis was accomplished by ABAQUS to predict the damage growth and material behavior of glass-fiber-reinforced polyurethane foam (RPUF). Wang et al. [22] applied a damage-modified ZWT nonlinear viscoelastic constitutive model to LS-DYNA, which was used to simulate the damage and failure of a windshield under a bird strike effectively. 

This paper chose liquid nitrile rubber (LNBR) to modify the epoxy resin. Pure epoxy resin and two kinds of LNBR/epoxy composites with different LNBR mass fractions were considered as the test materials. Quasi-static and dynamic compression experiments were performed to study the influence of LNBR on the mechanical properties of epoxy resin. Besides, the standard ZWT nonlinear viscoelastic model was chosen to predict the elastic behavior of LNBR/epoxy composites under wide ranges of strain rates and a set of model parameters for the tested materials were also obtained based on the integral form of ZWT constitutive equation. Afterwards, the ZWT model was implemented into LS-DYNA by user defined material (UMAT) depending on the differential constitutive equation. Finally, the simulation-test contrast verification of the algorithm had been carried out.

## 2. Specimen Fabrications

Bicomponent epoxy resin 2002A/B, obtained from the SLONT Company (Beijing, China), was chosen as the matrix material and LNBR, obtained from the Zeon Company (Tokyo, Japan), was used as a modifier to prepare the samples. The masses of LNBR and Epoxy 2002A were weighed separately and mixed together at a speed of 80 RPM for 10 min. The blended mixture was placed in a drying oven at 40 °C for 12 h. Then a certain amount of hardener 2002B was added to the mixture and blended together at 100 RPM for 10 min and the mixture was placed in a vacuum environment for 20 min to defoam. Afterwards, the resulting mixture was slowly poured into Polytetrafluoroethylene (PTFE) molds (designed by ourselves) which were covered by releasing agent and cured at 40 °C for 24 h. Finally, the specimens were removed from the molds and polished with 600-grid sandpaper to obtain smooth surfaces. 

Three material ratios were prepared for this study. M0 was a pure epoxy resin sample without LNBR, while M1, M2 were epoxy samples with a different wt % of LNBR. The detailed proportions of these samples are listed in Table 1.

## 3. Experiments

### 3.1. Quasi-Static Compression Experiments

Quasi-static compression experiments under strain rates ranging from 0.0001/s to 0.001/s were carried out at room temperature on a universal testing machine WDW300, which was made by the Changchun Kexin Test Instrument Company. The testing range of the load cell on the machined was 300 kN and the accuracy could reach 0.5%. A noncontact precision measurement video gauge was used to measure the deformation of the specimens. For the uniaxial compression tests, the strain rate is proportional to the compression speed in the form of

(1) $$\dot{\varepsilon }=\frac{\Delta \varepsilon }{\Delta t}=\frac{\Delta l/l_{0}}{\Delta t}=\frac{v}{l_{0}}$$

where Δt is the unit time, $l_{0}$ is the original gauge length of the specimen, Δl is the measured variation of the gauge length and *v* is the compression speed. The nominal stress is calculated via

(2) $$\sigma_{N}=\frac{F}{A_{0}}$$

where *F* is the force measured by the load cell and *A*_0_ is the initial cross-sectional area of the specimen. The average nominal strain in specimens could be obtained by

(3) $$\varepsilon_{N}=\frac{\Delta l}{l_{0}}$$

As the specimen size is constantly changing during the compression testing, the authors use the true stress and true strain in this paper. The true stress and true strain can be calculated by

(4) $$\sigma_{T}=\sigma_{N}\left(1-\varepsilon_{N}\right)$$

(5) $$\varepsilon_{T}=-\ln\left(1-\varepsilon_{N}\right)$$

**Figure 1:**
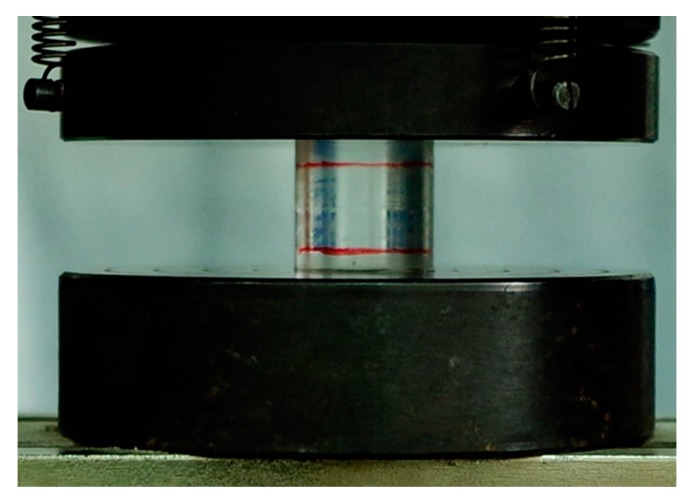
A specimen under quasi-static compression.

A specimen for the quasi-static compression experiment is shown in Figure 1. The diameter and height of the sample for quasi-static compression tests were both 28 mm. The two parallel lines were used for strain measurement. The distance between those lines decreased with the deformation of specimens, which could be captured by a noncontact measurement video gauge. According to Equation (1), the loading speed of the test machine was controlled by the specimen height and the required strain rate.

### 3.2. SHPB Compression Experiments

The split Hopkinson pressure bar is a typical dynamic experimental setup which has been widely used to investigate the dynamic mechanical behavior of materials [23]. In the present study, the SHPB system was also adopted for high-strain rate tests. The apparatus consisted of a projectile, an input bar and an output bar. 

The wave impedance of the specimens ranged from 2 × 10^6^ to 5 × 10^6^ kg/(m^2^·s) and the wave impedance of aluminum was 13 × 10^6^ kg/(m^2^·s). Although there was a gap between them, the stress conditions at both ends of the specimen could still be balanced. Besides, the wave impedance of the steel was much larger than that of the specimens, while the attenuation and dispersion phenomena should be considered during the wave propagation in the polymer bars, these two kinds of bars are not suitable for the epoxy resin samples. Hence, the projectile and all the bars were aluminum to match the wave impedance of the specimens. The length of the projectile was 400 mm, while the input and output bars were 2000 mm. The diameter of the projectile and bars were 37 mm to avoid the transverse inertia effect. The diameter of specimens for SHPB experiments was 25 mm, while the length was 4 mm. The projectile was driven by gas stored in the chamber to impact the free end of the input bar, then a longitudinal compression incident wave *ε_i_*(*t*) was generated. Once this wave reached the input bar-specimen interface, a part of it reflected back into the input bar and was named the reflected wave *ε_r_*(*t*). The other portion went through the specimen and the specimen-output bar interface, which generated the transmitted wave *ε_t_*(*t*). Two groups of strain gages were placed in the middle of the input and output bars. The strain gauges were connected to a dynamic strain indicator for signal conditioning and amplification. Meanwhile, the incident, reflected and transmitted waves were recorded by an oscilloscope.

According to the elastic wave propagation theory and the superposition principle, the stress and particle velocity associated with a single wave could be calculated from the associated strain measured by the strain gages. The forces and velocities at both end faces of the specimen were calculated by:

(6a) $$F_{input}\left(t\right)=A_{B}E_{B}\left(\varepsilon_{i}\left(t\right)+\varepsilon_{r}\left(t\right)\right)$$

(6b) $$F_{output}\left(t\right)=A_{B}E_{B}\varepsilon_{t}\left(t\right)$$

(6c) $$V_{input}\left(t\right)=C_{0}\left(\varepsilon_{i}\left(t\right)-\varepsilon_{r}\left(t\right)\right)$$

(6d) $$V_{output}\left(t\right)=C_{0}\varepsilon_{t}\left(t\right)$$

where *A_B_*, *E_B_* and *C*_0_ are the cross-sectional area, Young’s modulus and elastic wave speed of the bars, respectively. The change in the length of the specimen could be calculated from

(7) $$\Delta l=\int_{0}^{t}\left(V_{input}\left(\tau \right)-V_{output}\left(\tau \right)\right)d\tau$$

Based on the one-dimensional simple wave assumption, the stress, strain and strain rate could be obtained by the following formulas:

(8a) $$\sigma \left(t\right)=\frac{F_{input}+F_{output}}{2A_{0}}=\frac{A_{B}E_{B}\left(\varepsilon_{i}\left(t\right)+\varepsilon_{r}\left(t\right)+\varepsilon_{t}\left(t\right)\right)}{2A_{0}}$$

(8b) $$\varepsilon \left(t\right)=\frac{\Delta l}{l}=\frac{C_{0}}{l}\int_{0}^{t}\left(\varepsilon_{i}\left(\tau \right)-\varepsilon_{r}\left(\tau \right)-\varepsilon_{r}\left(\tau \right)\right)d\tau$$

(8c) $$\dot{\varepsilon }\left(t\right)=\frac{d\varepsilon \left(t\right)}{dt}=\frac{C_{0}}{l}\left(\varepsilon_{i}\left(\tau \right)-\varepsilon_{r}\left(\tau \right)-\varepsilon_{r}\left(\tau \right)\right)$$

**Figure 2:**
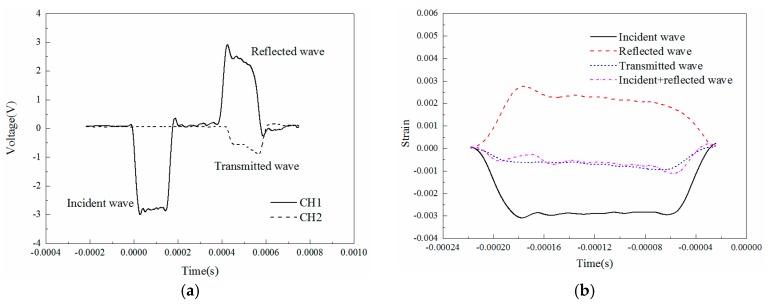
Typical SHPB wave forms for specimen. (**a**) Original voltage signals measured by strain gauges; (**b**) strain signals transformed from voltage signals.

The typical incident, reflected and transmitted waves in the experiments are shown in Figure 2. Figure 2a shows the original voltage signals measured by the strain gauges and Figure 2b presents the strain signals transformed from voltage signals.

## 4. Experimental Results and Discussion

### 4.1. Quasi-Static Experimental Results

#### 4.1.1. Stress-Strain Relationship

**Figure 3:**
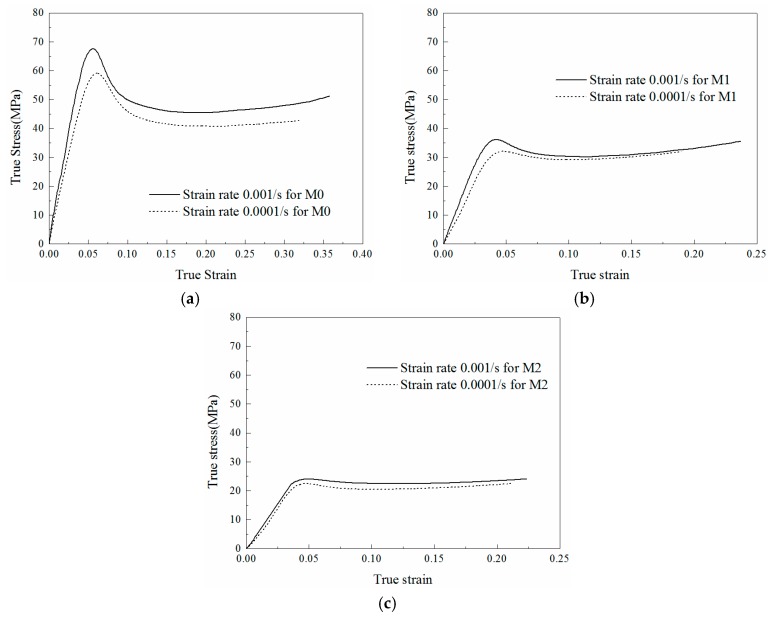
Comparison of compression stress versus compression strain plots under quasi-static loading for epoxy resin specimens with different content of LNBR: (**a**) 0% (M0); (**b**) 10% (M1); (**c**) 25% (M2).

The quasi-static compression experiments were performed under two strain rates (0.0001/s and 0.001/s) in this paper. In order to ensure the accuracy of test data, each experiment was repeated at least three times. The average data of the curves that repeated well was determined as the final results. The stress-strain curves under different strain rates for M0, M1 and M2 are presented in Figure 3.

It is obvious from Figure 3a that the strength of the material increases with the increasing strain rate, which is known as direct strain rate effect. Comparing Figure 3a with Figure 3b,c, it can be concluded that the strength of the specimen decreases with the enhancement of LNBR content under the same strain rate. Moreover, when the content of LNBR increases, the strain softening behavior in the plastic region is weakened and the plastic region also becomes flatter.

#### 4.1.2. Energy Absorption Analysis

**Figure 4:**
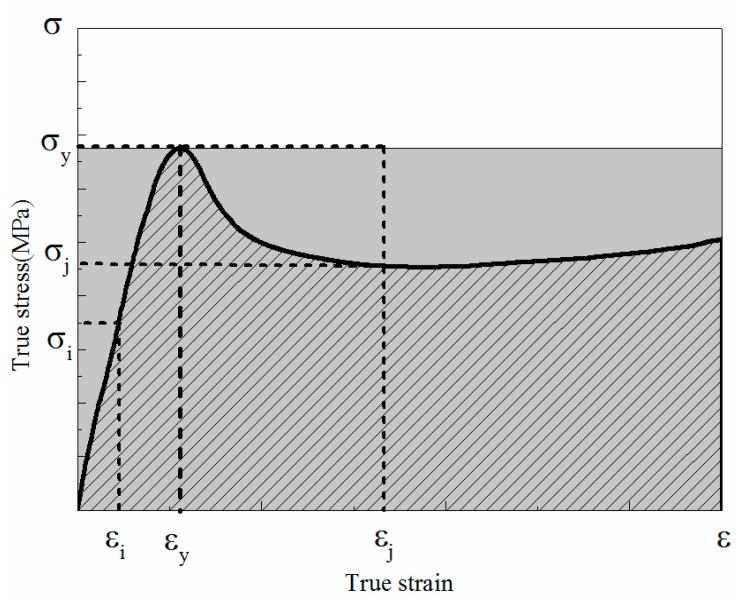
Energy absorption calculating process.

Epoxy resin is widely used as an electronic protective material, which can be adopted for energy absorption. Thus, the energy absorption capability of the tested materials is worth of discussing. The energy absorption per unit volume can be expressed as [24]

(9) $$W=\int_{0}^{\varepsilon }\sigma \left(\varepsilon \right)d\varepsilon$$

which is determined by the area under the stress-strain curves, namely, the area of oblique line in Figure 4. W is the energy absorption per unit volume, ε is the compression strain and σ represents the compression stress which is related to a function of ε. The energy absorption efficiency is defined by

(10) $$\eta =\frac{\int_{0}^{\varepsilon }\sigma \left(\varepsilon \right)d\varepsilon }{\sigma_{\max}\varepsilon }$$

where $\sigma_{\max}$ is the maximum stress experienced over the strain region. The parameter η is the ratio of energy absorption W to the product of the maximum stress $\sigma_{\max}$ and the current strain ε. The physical meaning of the energy absorption efficiency η can also be explained by the ratio of the area filled by oblique line to the grey filled area in Figure 4. It can be observed that, the maximum stress is $\sigma_{\max}=\sigma_{i}$ when $\varepsilon_{i}<\varepsilon_{y}$; and the maximum stress is $\sigma_{\max}=\sigma_{y}$ when $\varepsilon_{j}\geq \varepsilon_{y}$.

**Figure 5:**
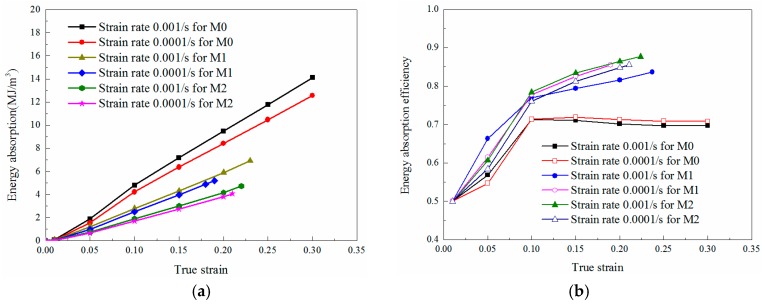
Energy absorption performance of epoxy resin specimens with different content of LNBR: 0% (M0), 10% (M1), 25% (M2) under quasi-static strain rates. (**a**) Energy absorption; (**b**) energy absorption efficiency.

The total energy absorption and energy absorption efficiencies of different materials under quasi-static loadings are plotted in Figure 5. For the same material, a slight difference can be observed from the efficiency plots under different quasi-static strain rates, as the tendency and the maximum value are both similar. Under the same strain condition, the energy absorbed by M0 is the largest but the tendency of energy efficiency is opposite. Namely, the addition of LNBR decreases the energy absorption but increases the energy absorption efficiency of the epoxy resin. This phenomenon can be explained by that the LNBR reduces the strength as well as the strain softening behavior of the epoxy resin, which can also be reflected from the stress-strain curves in Figure 3.

### 4.2. SHPB Experimental Results

#### 4.2.1. Dynamic Stress-Strain Relationship

**Figure 6:**
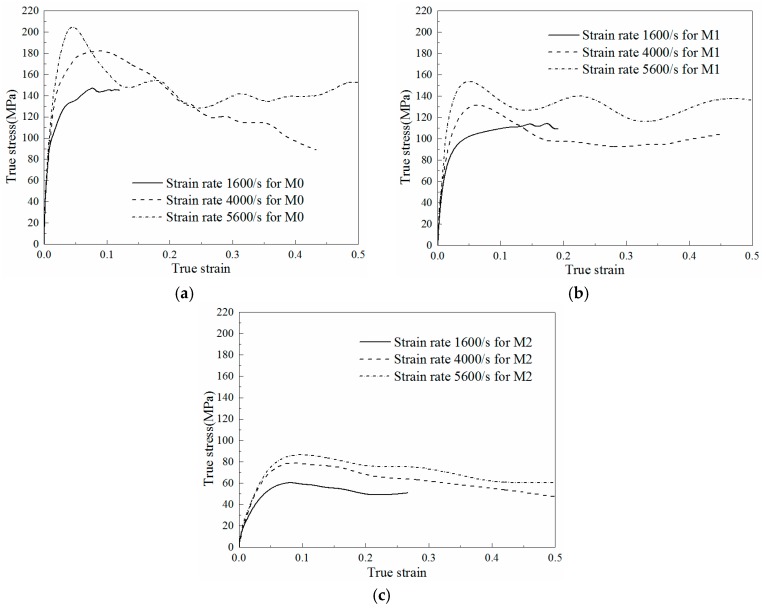
Dynamic stress-strain curves of three specimens with different LNBR content: (**a**) 0% (M0); (**b**) 10% (M1); (**c**) 25% (M2) at different strain rates.

The projectile velocities of SHPB experiments were 10 m/s, 20 m/s and 30 m/s, which leads to the relevant strain rates calculated from Equation (8c) of 1600/s, 4000/s and 5600/s respectively. Each dynamic experiment was repeated at least three times, which was similar to the quasi-static condition. The average stress-strain curves of M0, M1 and M2 under different loading rates are plotted in Figure 6a–c, respectively.

Some non-negligible differences can be observed from Figure 6a–c. Compared with M1 (Figure 6b), the strain-softening phenomenon in M0 (Figure 6a) is more visible. Nevertheless, the stress in the plastic region of M2 (Figure 6c) decreases with the increasing of strain and the strain hardening effect is absent all the time.

#### 4.2.2. Strain Rate Effect

**Figure 7:**
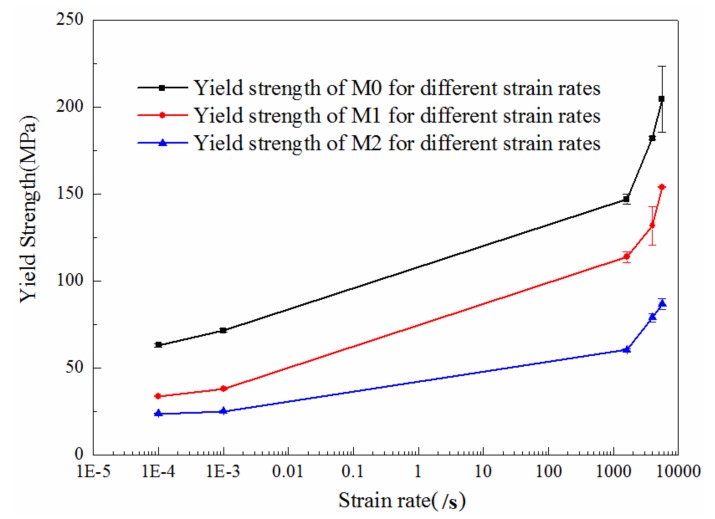
Yield strengths of specimens with different LNBR content: 0% (M0), 10% (M1), 25% (M2) under different strain rates.

It has been widely reported that the materials exhibit distinct properties especially for the yield strengths under dynamic loadings when compared with their quasi-static responses. The yield strengths of the three materials tested in this manuscript under different strain rates are presented in Table 2 and Figure 7 and the corresponding error range of the strengths are also included. It can be noted that the maximum error is smaller than 10%, which demonstrates the high repeatability of the experiments. The results exhibit that the dynamic yield strength decreases with the increase of LNBR content under the same strain rate and the direct strain rate effect can also be observed, which are similar to the quasi-static results. Apparently, the dynamic yield strengths of the three materials are all higher than their quasi-static yield strengths.

#### 4.2.3. Dynamic Deformation Observation

**Figure 8:**
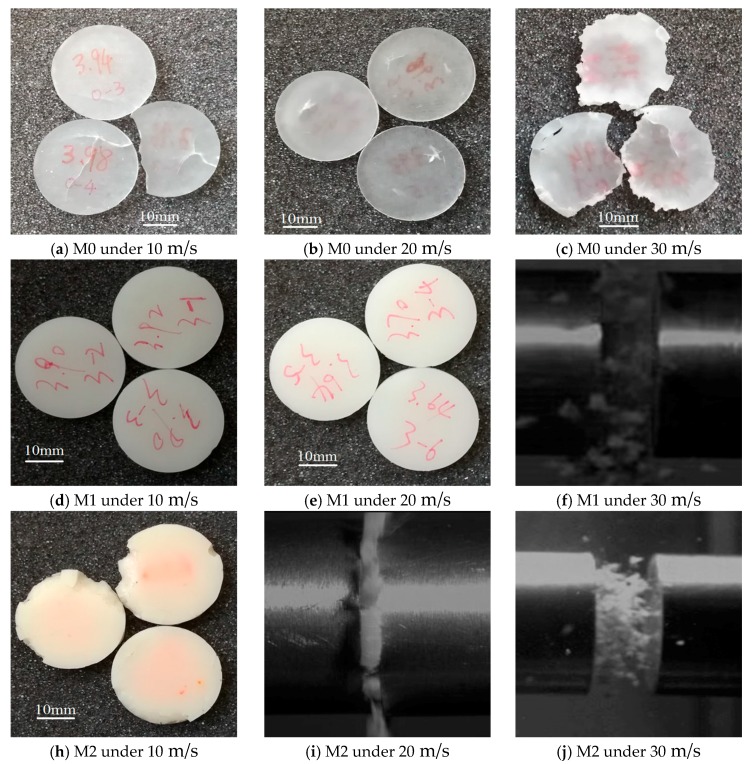
Final shape of specimens with different LNBR content at different impact loading: (**a**–**e**,**g**) show the recovered samples with high integrity; (**f**–**i**) show the moment when the specimens crushed into powder.

In order to reveal the deformation mechanism of M0, M1 and M2, the final shapes of the specimens are exhibited in Figure 8a–e,g. However, some specimens were crushed into powder and intact samples could not be recovered after experiments. Thus, the crush moments of the corresponding specimens were captured by high speed photography which are presented in Figure 8f,h,i. It can be easily observed from these images that the specimens of M2 are the most crumbly, which can explain the continuous strain-softening effect on the corresponding stress-strain curves in Figure 6c.

The final shapes of the M0 specimens presented in Figure 8a–c reveal a special phenomenon. The specimens shown in Figure 8a exhibit several long cracks and breakage under an impact speed of 10 m/s, while serious damage and cracks can be observed in the specimens under an impact speed of 30 m/s, as presented in Figure 8c. However, only tiny blooming shape cracks can be observed in the specimens under an impact speed of 20 m/s (Figure 8b). This may be explained by that a temperature enhancement occurred in the specimens of M0 under 20 m/s, which reduces the brittleness of the material. Moreover, a continuous softening phenomenon in the plastic region of the stress-strain curve under 20 m/s is also caused by the thermal effect, which does not appear in the stress-strain curves of 10 m/s and 30 m/s. This may be resulted from that the lowest impact speed of 10 m/s does not cause a temperature rise before the long cracks emerge, while the highest impact speed of 30 m/s leads to an adiabatic effect as the compression time is too short to heat the specimens up.

Figure 8a,d,g exhibit the specimens of M0, M1 and M2 under an impact speed of 10 m/s respectively. It can be observed that only the specimens of M1 have a complete appearance without visible damage, while the specimens of M0 (Figure 8a) have several through cracks and the specimens of M2 (Figure 8g) show small breakages. The integrity of M1 is the best at an impact speed of 10 m/s. According to Figure 8b,e,h, both the specimens of M0 and M1 remain complete external shapes, which indicate that the integrity of M1 is as good as M0 at an impact speed of 20 m/s. However, as the LNBR decreases the strength of epoxy resin, the specimens of M1 and M2 are both crushed into powder during the impact with 30 m/s. The specimens of M0 also exhibit obvious damage under the same condition but remnants could still be found after the experiments. All the above analyses indicate that 10% LNBR content can enhance the integrity of specimens at the impact speed of 10 m/s and 20 m/s. Nevertheless, it should also be noted that too much LNBR makes specimens crushable.

## 5. Model Calibration and Parameter Identification

### 5.1. Viscoelastic Material Models

**Figure 9:**
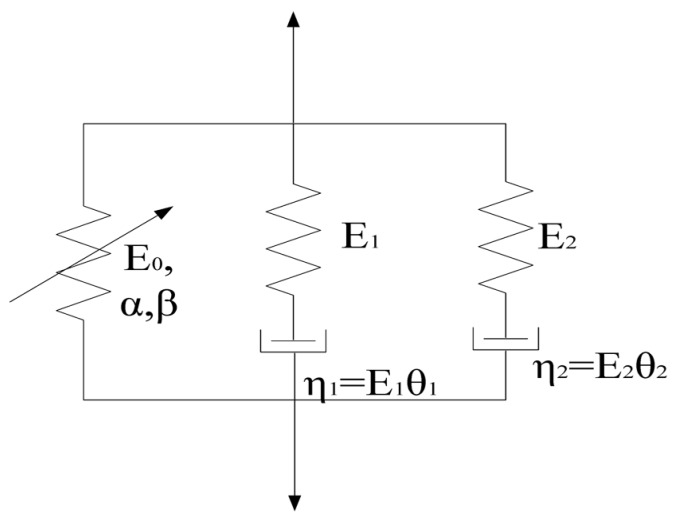
Rheological form of the ZWT model.

The rate-dependent behavior is a very important and well-known mechanical property of polymeric material. The ZWT nonlinear viscoelastic model, which is composed of a nonlinear spring, a low strain rate Maxwell viscoelastic element I and a high strain rate Maxwell viscoelastic element II, can precisely describe their mechanical behavior within the elastic deformation under strain rates magnitude of 0.0001/s–1000/s [25]. Figure 9 displays the rheological form of the model and the constitutive equation is expressed as

(11) $$\sigma =\sigma_{e}+\sigma_{r_{1}}+\sigma_{r_{2}}$$

where σ denotes the total stress, $\sigma_{e}$ describes the nonlinear elastic response, $\sigma_{r1}$ represents the linear Maxwell viscoelastic response at low strain rates, $\sigma_{r2}$ represents the linear Maxwell viscoelastic response at high strain rates.

The nonlinear elastic response can be indicated as

(12) $$\sigma_{e}=E_{0}\varepsilon +\alpha \varepsilon^{2}+\beta \varepsilon^{3}$$

where ε denotes the strain, $E_{0}$, α, and β are the elastic constants.

The Maxwell viscoelastic responses at low strain rate can be represented in integral and differential forms [26]. The integral form is shown as

(13a) $$\sigma_{r_{1}}=E_{1}\int_{0}^{t}\dot{\varepsilon }\left(t\right)\exp\left(-\frac{t-\tau }{\theta_{1}}\right)d\tau$$

and the differential form is shown as

(13b) $$\frac{\partial \sigma_{r_{1}}}{\partial t}=E_{1}\frac{\partial \varepsilon }{\partial t}-\frac{\sigma_{r_{1}}}{\theta_{1}}$$

where $E_{1}$ and $\theta_{1}$ are the elastic constant and relaxation time of low strain rate Maxwell element I, $\eta_{1}=E_{1}\theta_{1}$ is the corresponding viscosity coefficient.

Similarly, the integral and differential forms of Maxwell viscoelastic responses at high strain rate can be represented as

(14a) $$\sigma_{r_{2}}=E_{2}\int_{0}^{t}\dot{\varepsilon }\left(t\right)\exp\left(-\frac{t-\tau }{\theta_{2}}\right)d\tau$$

(14b) $$\frac{\partial \sigma_{r_{2}}}{\partial t}=E_{2}\frac{\partial \varepsilon }{\partial t}-\frac{\sigma_{r_{2}}}{\theta_{2}}$$

where $E_{2}$ and $\theta_{2}$ are the elastic constant and relaxation time of high strain rate Maxwell element II, $\eta_{2}=E_{2}\theta_{2}$ is the corresponding viscosity coefficient.

Hence, the constitutive equation of the ZWT viscoelastic model can be expressed in two forms. The integral constitutive equation is composed of Equation (12), (13a) and (14a), which is expressed as follows.

(15) $$\sigma =E_{0}\varepsilon +\alpha \varepsilon^{2}+\beta \varepsilon^{3}+E_{1}\int_{0}^{t}\dot{\varepsilon }\left(t\right)\exp\left(-\frac{t-\tau }{\theta_{1}}\right)d\tau +E_{2}\int_{0}^{t}\dot{\varepsilon }\left(t\right)\exp\left(-\frac{t-\tau }{\theta_{2}}\right)d\tau$$

Combining Equation (12), Equation (13b) with Equation (14b), the differential form of constitutive Equation (11) is expressed as follows.

(16) $$\frac{\partial \sigma }{\partial t}=\frac{\partial \sigma_{e}}{\partial \varepsilon }\frac{\partial \varepsilon }{\partial t}+E_{1}\frac{\partial \varepsilon }{\partial t}-\frac{\sigma_{r_{1}}}{\theta_{2}}+E_{2}\frac{\partial \varepsilon }{\partial t}-\frac{\sigma_{r_{2}}}{\theta_{2}}$$

### 5.2. Parameter Identification

Under quasi-static compression conditions, the high strain rate Maxwell element II is totally relaxed at the beginning of loading, which means that Equation (14a) equals zero. Then the constitutive Equation (15) can be reduced to

(17) $$\sigma =E_{0}\varepsilon +\alpha \varepsilon^{2}+\beta \varepsilon^{3}+E_{1}\int_{0}^{t}\dot{\varepsilon }\left(t\right)\exp\left(-\frac{t-\tau }{\theta_{1}}\right)d\tau$$

Conversely, there is no enough time for the low strain rate Maxwell element I to relax until the end of loading under the dynamic compression conditions, which can be regarded as a single spring element with an elastic constant $E_{1}$. Consequently, the constitutive Equation (15) can be reduced to

(18) $$\sigma =E_{0}\varepsilon +\alpha \varepsilon^{2}+\beta \varepsilon^{3}+E_{1}\varepsilon +E_{2}\int_{0}^{t}\dot{\varepsilon }\left(t\right)\exp\left(-\frac{t-\tau }{\theta_{2}}\right)d\tau$$

**Figure 10:**
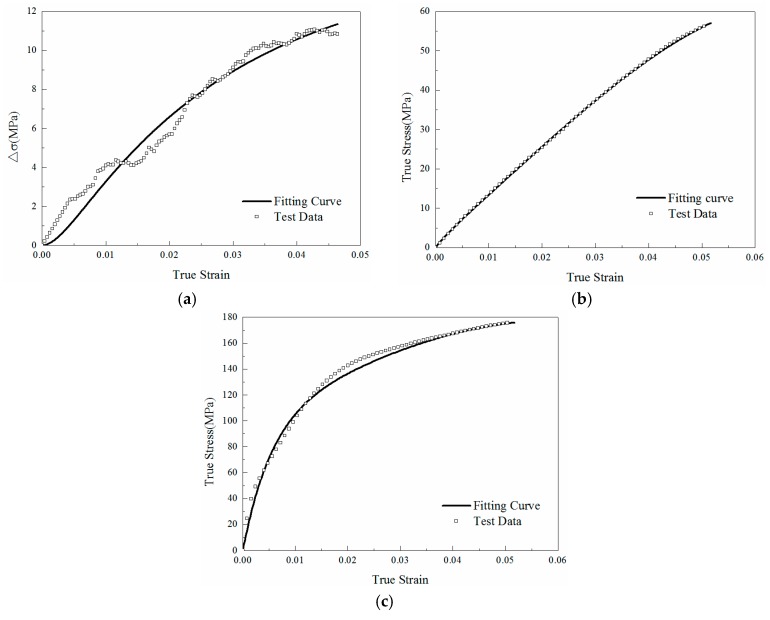
Fitting results of ZWT model parameters for pure epoxy resin (M0): (**a**) fitting results of difference curves Δ*σ* for *E*_1_ and *θ*_1_; (**b**) fitting results of quasi-static strain rate (0.001/s) test data for *E*_0_, *α* and *β*; (**c**) fitting results of high strain rate(4000/s) test data for *E*_2_ and *θ*_2_.

Thus, the curves in Figure 3 and Figure 6 can be described by Equations (17) and (18) respectively. The difference between the two quasi-static curves in Figure 3 can be reflected by the following equation [17]

(19) $$\begin{array}{l} \Delta \sigma =E_{1}\int_{0}^{\varepsilon /{\dot{\varepsilon }}_{1}}{\dot{\varepsilon }}_{1}\exp\left(-\frac{\varepsilon /{\dot{\varepsilon }}_{1}-\tau }{\theta_{1}}\right)d\tau -E_{1}\int_{0}^{\varepsilon /{\dot{\varepsilon }}_{2}}{\dot{\varepsilon }}_{2}\exp\left(-\frac{\varepsilon /{\dot{\varepsilon }}_{2}-\tau }{\theta_{1}}\right)d\tau \\ =E_{1}\theta_{1}{\dot{\varepsilon }}_{1}\left(1-\exp\left(-\frac{\varepsilon }{{\dot{\varepsilon }}_{1}\theta_{1}}\right)\right)-E_{1}\theta_{1}{\dot{\varepsilon }}_{2}\left(1-\exp\left(-\frac{\varepsilon }{{\dot{\varepsilon }}_{2}\theta_{1}}\right)\right) \end{array}$$

where ${\dot{\varepsilon }}_{1}$ and ${\dot{\varepsilon }}_{2}$ are the two different quasi-static strain rates (${\dot{\varepsilon }}_{1}$ > ${\dot{\varepsilon }}_{2}$). The difference Δσ is obtained by subtracting the values of the experimental curves at a strain rate of ${\dot{\varepsilon }}_{1}$ = 0.001/s and a strain rate of ${\dot{\varepsilon }}_{2}$ = 0.0001/s. During curve fitting in this study, a Levenberg-Marquardt algorithm was employed to minimize the difference between the experimental data and the equation prediction. Firstly, $E_{1}$ and $\theta_{1}$ were acquired by fitting the experimental data Δσ to Equation (19). Afterwards, the quasi-static parameters $E_{0}$, α and β were obtained by fitting the stress-strain curve under the strain rate of 0.001/s to Equation (17), while $\sigma_{m}$, m and n can be obtained from the data as well. Finally, the high strain rate response parameters $E_{2}$ and $\theta_{2}$ can be obtained by fitting the test data under 4000/s to Equation (18). The contrasting results of those processes for M0 are shown in Figure 10. Similarly, the complete parameters of M1 and M2 can also be obtained from the above methods. All parameters are listed in Table 3.

### 5.3. Prediction of Compression Behavior under Other Strain Rates

**Figure 11:**
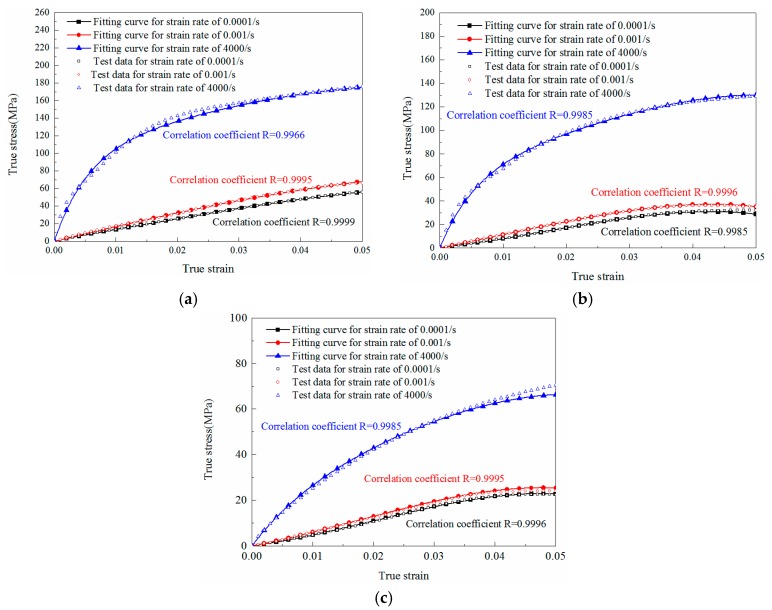
Comparison between model fitting results and experimental data at different strain rates for specimens with different LNBR content: (**a**) 0% (M0); (**b**) 10% (M1); (**c**) 25% (M2).

**Figure 12:**
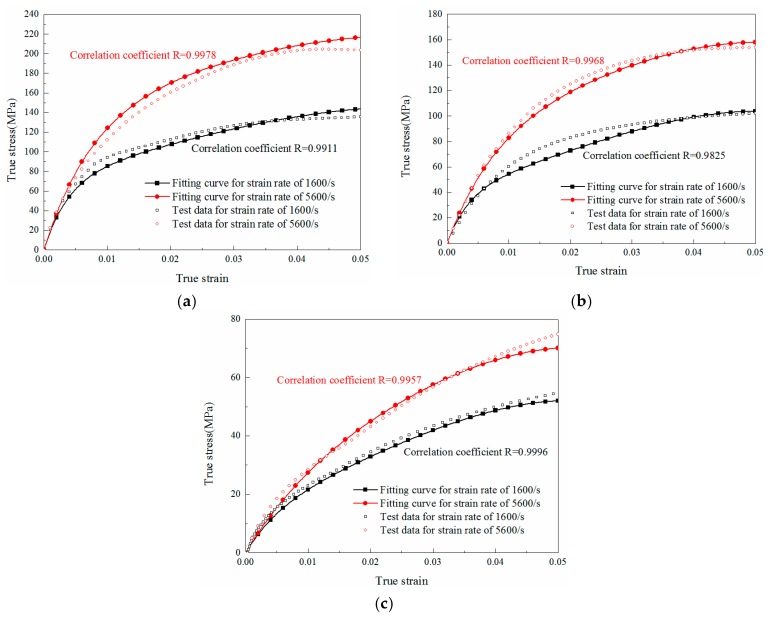
Comparison between predicted model results and experimental data at different strain rates for specimens with different LNBR content: (**a**) 0% (M0); (**b**) 10% (M1); (**c**) 25% (M2).

The experimental curves and relevant ZWT model fitting results of M0, M1 and M2 under different strain rates are shown in Figure 11. To verify the accuracy of those parameters, the comparisons between experimental results and predicted curves fitted by ZWT model parameters under strain rates of 1600/s and 5600/s are shown in Figure 12. It can be concluded that the predicted curves also match well with the experimental results of these materials. Additionally, the correlation coefficients R between the fitting curves and experimental results are listed next to the curves in Figure 11 and Figure 12, which demonstrate the validity of the model. Hence, the ZWT nonlinear viscoelastic model can be employed to describe the viscoelastic mechanical properties of all the three materials (M0, M1 and M2) over a wide range of strain rates.

## 6. FE Simulations

Since the ZWT constitutive model could describe the mechanical behavior of epoxy resin in elastic region under different strain rates, it also makes sense to extend this constitutive model to simulate the material response by finite element method. This section introduces an algorithm which could implement the ZWT model into LS-DYNA by using of the user-defined material subroutine (UMAT) and a simple comparison between the algorithm and experimental results is also presented. 

### 6.1. Incremented Algorithm of ZWT Constitutive Model in the Finite Element Analysis

The incremental constitutive equation of ZWT viscoelastic model can be deduced from Equation (16) easily, which is represented as follows.

(20) $$\Delta \sigma \left(t+\Delta t\right)=\left[E_{0}+2\alpha \varepsilon \left(t\right)+3\beta \varepsilon^{2}\left(t\right)+E_{1}+E_{2}\right]\Delta \varepsilon \left(t+\Delta t\right)-\left(\frac{\sigma_{r1}\left(t\right)}{\theta_{1}}+\frac{\sigma_{r2}\left(t\right)}{\theta_{2}}\right)\Delta t$$

Based on Equations (13b) and (14b), the stress increments of Maxwell element I and Maxwell element II can be expressed as:

(21) $$\Delta \sigma_{r1}\left(t+\Delta t\right)=E_{1}\Delta \varepsilon \left(t+\Delta t\right)-\frac{\sigma_{r1}\left(t\right)}{\theta_{1}}\Delta t$$

(22) $$\Delta \sigma_{r2}\left(t+\Delta t\right)=E_{2}\Delta \varepsilon \left(t+\Delta t\right)-\frac{\sigma_{r2}\left(t\right)}{\theta_{2}}\Delta t$$

In three dimensions, the above formulae can be rewritten as follows:

(23) $$\Delta \sigma_{ij}\left(t+\Delta t\right)=\left[E_{0}+2\alpha \varepsilon_{ij}\left(t\right)+3\beta \varepsilon^{2}{}_{ij}\left(t\right)+E_{1}+E_{2}\right]A_{ijkl}\Delta \varepsilon_{kl}\left(t+\Delta t\right)-\left(\frac{\sigma_{ij,r1}\left(t\right)}{\theta_{1}}+\frac{\sigma_{ij,r2}\left(t\right)}{\theta_{2}}\right)\Delta t$$

(24) $$\Delta \sigma_{ij,r1}\left(t+\Delta t\right)=E_{1}A_{ijkl}\Delta \varepsilon_{kl}\left(t+\Delta t\right)-\frac{\sigma_{ij,r1}\left(t\right)}{\theta_{1}}\Delta t$$

(25) $$\Delta \sigma_{ij,r2}\left(t+\Delta t\right)=E_{2}A_{ijkl}\Delta \varepsilon_{kl}\left(t+\Delta t\right)-\frac{\sigma_{ij,r2}\left(t\right)}{\theta_{2}}\Delta t$$

According to Equations (13a) and (14a), the three-dimensional expression of $\sigma_{ij,r1}$ and $\sigma_{ij,r2}$ can be represented as

(26) $$\sigma_{ij,r1}\left(t\right)=E_{1}\theta_{1}A_{ijkl}\frac{\Delta \varepsilon_{kl}\left(t\right)}{\Delta t}\left(1-\exp\left(-\frac{t}{\theta_{1}}\right)\right)$$

(27) $$\sigma_{ij,r2}\left(t\right)=E_{2}\theta_{2}A_{ijkl}\frac{\Delta \varepsilon_{kl}\left(t\right)}{\Delta t}\left(1-\exp\left(-\frac{t}{\theta_{2}}\right)\right)$$

where

(28) $$A_{ijkl}=\frac{1}{\left(1+v\right)\left(1-2v\right)}\left[\begin{array}{cccccc} 1-v & v & v & 0 & 0 & 0\\ v & 1-v & v & 0 & 0 & 0\\ v & v & 1-v & 0 & 0 & 0\\ 0 & 0 & 0 & \frac{1-2v}{2} & 0 & 0\\ 0 & 0 & 0 & 0 & \frac{1-2v}{2} & 0\\ 0 & 0 & 0 & 0 & 0 & \frac{1-2v}{2} \end{array}\right]$$

in which $A_{ijkl}$ is elasticity matrix and v is Poisson’s ratio.

Thus, the recurrence relation of stress can be calculated by

(29) $$\sigma_{ij}\left(t+\Delta t\right)=\sigma_{ij}\left(t\right)+\Delta \sigma_{ij}\left(t+\Delta t\right)$$

**Figure 13:**
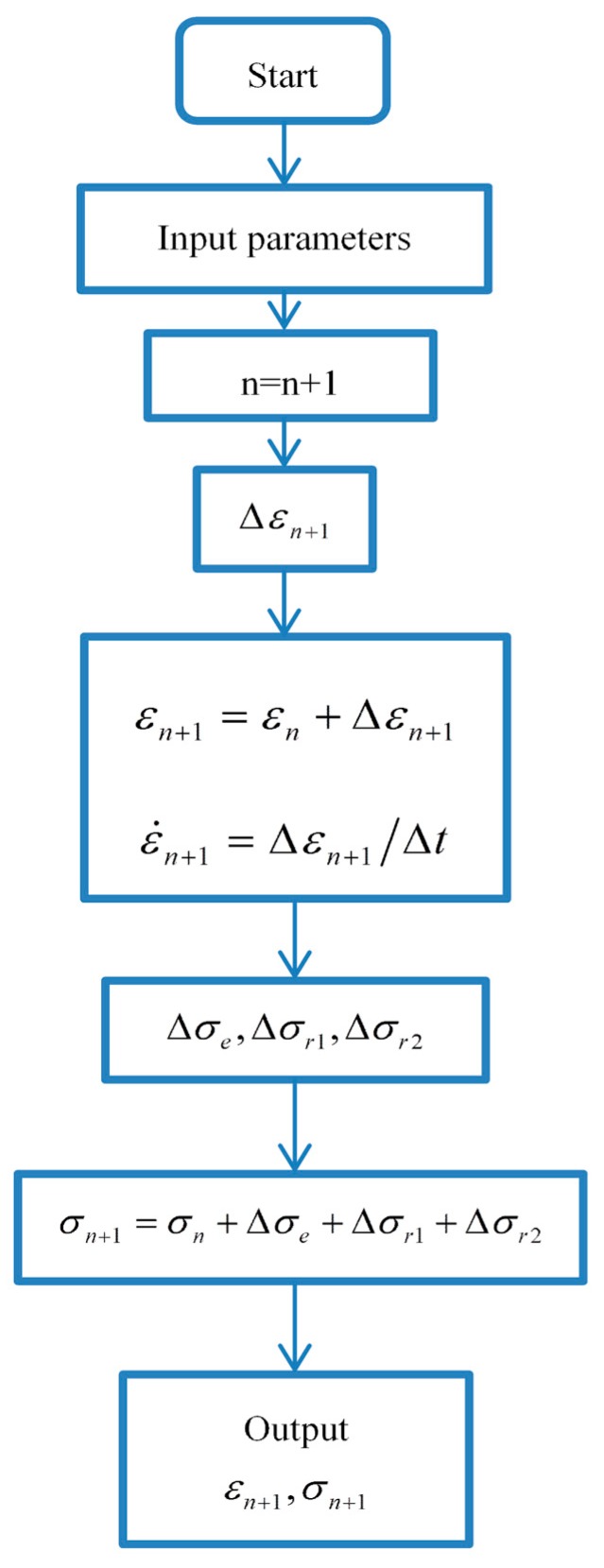
Flowchart of the UMAT subroutine.

After the above processes, Equation (29) can be implemented into LS-DYNA software by adopting the user-defined material (UMAT) subroutine and the flow chart of the UMAT subroutine is shown in Figure 13. Afterwards, the ZWT nonlinear viscoelastic model is available in LS-DYNA for dynamic simulation. 

### 6.2. Simulation Verification

**Figure 14:**
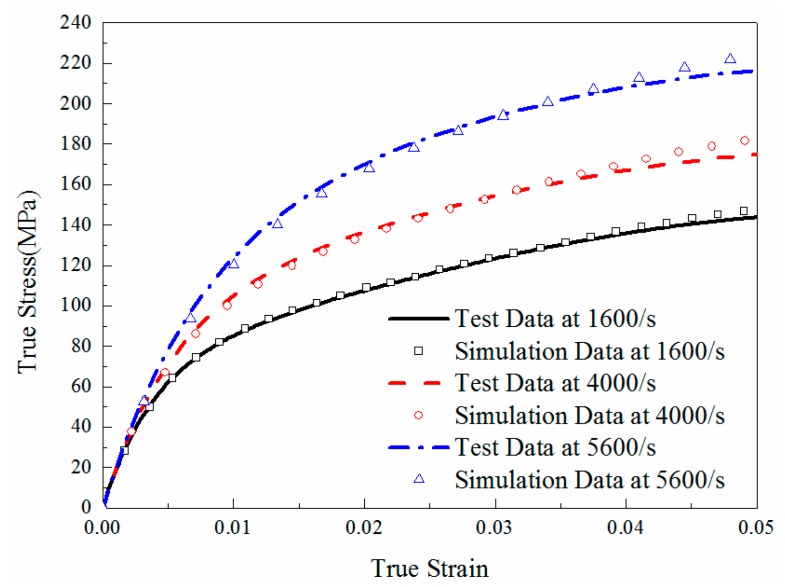
Comparison between simulation and test results of pure epoxy resin.

In order to verify the correctness of the algorithm derived in the previous section, a series of FE simulations which were calculated by the new algorithm had been conducted to compare with the experimental results. The comparison results are shown in Figure 14, which implies that the simulation results are in a good agreement with the experimental results. Therefore, this comparison results prove that the algorithm in this paper can be applied into LS-DYNA to describe the viscoelastic behaviors of epoxy resin.

## 7. Conclusions

In this study, the mechanical properties of epoxy resin with different content of LNBR were examined by quasi-static and dynamic compression experiments. The ZWT nonlinear viscoelastic model parameters for each material were fitted from the test data and this constitutive model was implemented into the user material program of LS-DYNA. The conclusions can be summarized as follows:

(1) The strain rate effect of epoxy resin has been observed. Not only the yield strength but also the elastic stiffness of epoxy resin enhances with the increasing of strain rate.
(2) The LNBR modifier can effectively increase the energy absorption efficiency and the strain-softening effect of the material decreases with increasing LNBR mass fractions. Meanwhile, the specimens with 10% LNBR additive can enhance their integrity under 10 m/s projectile impact.
(3) The yield strengths and elastic stiffness of the modified epoxy resins are reduced by the soft rubber additive, which are also embodied in the ZWT model parameters.
(4) The algorithm mentioned in this study can embed the ZWT constitutive model into the LS-DYNA software successfully, which would have many applications in the engineering.

## Figures and Tables

**Table 1:** Constituents of test materials.

Material Number	Mass/g			wt % of LNBR
	Epoxy 2002A	Hardener 2002B	LNBR	
M0	80	40	0	0%
M1	80	40	12	10%
M2	80	40	30	25%

**Table 2:** Quasi-static and high strain rate compression strength for M0, M1 and M2.

Material	0.0001/s	0.001/s	1600/s	4000/s	5600/s
M0	62.83 (±1.15)	71.45 (±0.8)	146.92 (±3)	181.97 (±1.1)	204.33 (±19)
M1	33.58 (±0.25)	37.65 (±0.4)	113.58 (±3)	131.46 (±11.1)	153.76 (±0.2)
M2	23.58 (±0.3)	25.04 (±0.3)	60.24 (±0.6)	78.84 (±2.5)	86.52 (±3)

**Table 3:** ZWT model parameters of test materials.

Material No.	*E*_0_ (GPa)	*α* (GPa)	*β* (GPa)	*E*_1_ (GPa)	*θ*_1_ (s)	*E*_2_ (GPa)	*θ*_2_ (μs)
M0	1.141	5.765	−136.834	0.573	27.8	19	1.38
M1	0.505	25.63	−490.71	0.637	11.17	12.01	1.46
M2	0.29	17.71	−291.58	0.219	13.7	3.05	2.19
